# Underwater Wireless Communications

**DOI:** 10.3390/s24217075

**Published:** 2024-11-03

**Authors:** Hamada Esmaiel, Haixin Sun

**Affiliations:** 1 Electrical Engineering Department, College of Engineering, King Khalid University, Abha 61411, Saudi Arabia; 2 Electrical Engineering Department, Faculty of Engineering, Aswan University, Aswan 81542, Egypt; 3 Department of Information and Communication, School of Informatics, Xiamen University, Xiamen 361005, China; hxsun@xmu.edu.cn

Effective underwater wireless communications (UWCs) are essential for a variety of military and civil applications, such as submarine communication and discovery of new natural resources in the underwater environment [1,2,3]. Researchers and industry have been striving to uncover the vast expanse of ocean water to enhance the environment. The Internet of Underwater Things (IoUTs) can be the desirable system for such tasks [1,2]. But the physical characteristics of the oceanic environments are still big challenges [3,4]. These environmental challenges restrict the recharging capabilities for IoUTs nodes and limit the underwater acoustic channel bandwidth [5,6]. These challenges have motivated researchers to efficiently design underwater wireless communication systems with effective energy and spectrum communication systems [7,8,9]. These challenges have motivated us to invite interested researchers to design highly efficient energy and spectral underwater wireless communication systems for inclusion in this Special Issue. The submitted papers in this Special Issue targeted new design schemes to solve the underwater wireless communication system and enable the Internet of Underwater Things.

The following Special Issue compiles 10 papers that offer a comprehensive overview of recent advancements in underwater wireless communication systems. The collection highlights key findings and their implications for future research and applications. This Special Issue includes studies that cover energy harvesting for IoUTs, physical data transmission over hydroacoustic channel, equalization and interference cancelation of the underwater continuous phase modulation (CPM) technique, long-range biomimetic covert underwater communications, propagation wave using magnetic coils for underwater data transmission, applications of vector sensor directional components in the underwater acoustic communication, using passive time reversal autoencoder for enhancing reliability of the underwater wireless data transmission, image super resolution-based channel estimation, and applying deep learning and artificial intelligence to improve the underwater communication.

Contribution 1 focuses on creating a dependable wireless underwater communication system and testing it in situations where there is non-line-of-sight (NLOS) propagation. They used diversity combining, which involves delivering a single bit on two carriers, as well as multiple frequency-shift keying (MFSK) modulation techniques. In a lab setting, they experimented with their technique, modeling underwater signal propagation throughout the wreck’s penetration. Using the correlation method, we were able to determine the impulse responses at specific measurement sites, which allowed us to analyze the propagation conditions. They also found the bit error rate (BER) to compare things in the same conditions using binary phase shift keying (BPSK) and direct sequence spread spectrum (DSSS) modulation. Findings showed that the method proposed in Contribution 1 for sending data wirelessly over a hydroacoustic channel in places without line of sight (LOS) worked. The findings lay the groundwork for creating a communication system with self-governing underwater vehicles that function in challenging propagation environments, like ports and wrecks.

In Contribution 2, researchers try to solve the intersymbol interference problem by proposing an algorithm for prefiltered single-carrier frequency-domain equalization. Their approach enhances the continuous-phase underwater modulation scheme’s bandwidth and power. In the same way, Contribution 3 suggests a better frequency domain turbo equalization method that uses iterative channel estimation and feedback to achieve good performance while also making the receiver simpler. Contribution 3 uses the proposed equalization scheme to enhance interference cancelation and eliminate the underwater cannel effect. Contribution 4 solves the multipath underwater channel effect by using a passive time reversal-autoencoder. Contribution 4 actively utilizes common synchronous signals in underwater acoustic communication as detection signals to achieve passive time reversal without external signals. We then design a passive time reversal-autoencoder to suppress multipath effects, enhance signals’ features, improve modulation recognition accuracy, and improve environmental adaptability. In contribution 5, the effect of vector sensor directional parts on steerability is measured and compared using receiver topologies based on beamforming and passive time reversal. This contribution describes an actual experiment in which a ship-hung acoustic source transmits coherent modulated communication signals at different ranges and from several directions. Contribution 6 proposes a biomimicking modulation technique for long-distance underwater acoustic communication. The proposed scheme represented the time-dependent frequency variation in the whales’ whistles and imitated the sound of a huge whale. The proposed scheme is utilized for long-range underwater wireless communication in order to overcome the significant multipath delay. Contribution 7 introduces a wireless communication system that uses magnetic coils for propagation, addressing the lack of acoustic wave propagation. Contribution 7 offers a mathematical model and a real experiment to demonstrate the reliability of magnetic coils for underwater data transmission, which includes the transceiver components. Based on 5G technologies, Contribution 8 provides non-orthogonal multiple access (NOMA) to be used in IoUTs physical communication. In contribution 8, time-switching simultaneous wireless information and power transfer (TS-SWIPT) is suggested as a way to obtain energy from power that is sent during the guard interval in the multicarrier schemes. The proposed energy harvesting scheme recovers energy from the wasted power during the long guard interval, thereby enhancing the energy efficiency of the NOMA time-division synchronization orthogonal frequency division multiplexing multicarrier system. By this way, Contribution 8 improves the total system throughput, energy efficiency, and harvested energy. Contribution 9 addresses the lack of traditional orthogonal frequency division multiplexing over underwater sound channels. It suggests orthogonal chirp division multiplexing (OCDM) that is based on deep learning. They derived a matrix completion problem from pilot-based channel estimation, which is mathematically analogous to the picture super-resolution problem that arises in image processing. A deep learning model performs joint signal-to-noise (SNR) training instead of using a conventional network with a single SNR. Contribution 10 discusses how packets transmitted over underwater wireless communication can be easily intercepted. The authors of this contribution discussed how to define the trust feature attributes and trust evaluation matrix for underwater network nodes. The authors also discussed how to create a trust evaluation model based on a generative adversarial network (GAN) and identify malicious nodes by creating a trust feature profile for each node. This makes it easier to identify malevolent nodes in underwater networks, especially in cases when the training data are imbalanced and lack labeling.

The aforementioned studies collectively advance the field of underwater wireless communication by using energy harvesting and Internet of Underwater Things technologies, physical layer data transmission over an underwater channel, deep learning approaches for underwater communication systems, and equalization techniques. We would like to express our gratitude to each and every author and reviewer whose work has enhanced this Special Issue.

## References

[1] Zeng Z., Fu S., Zhang H., Dong Y., Cheng J. (2016). A survey of underwater optical wireless communications. IEEE Commun. Surv. Tutor..

[2] Menaka D., Gauni S., Manimegalai C.T., Kalimuthu K. (2022). Challenges and vision of wireless optical and acoustic communication in underwater environment. Int. J. Commun. Syst..

[3] Junejo N.U.R., Sattar M., Adnan S., Sun H., Adam A.B., Hassan A., Esmaiel H. (2023). A survey on physical layer techniques and challenges in underwater communication systems. J. Mar. Sci. Eng..

[4] Mostafa M., Esmaiel H., Mohamed E.M. (2018). A Comparative Study on Underwater Communications for Enabling C/U Plane Splitting Based Hybrid UWSNs, Proceedings of the IEEE WCNC, Barcelona, Spain, 15–18 April 2018.

[5] Qasem Z.A., Wang J., Leftah H.A., Sun H., Hong S., Qi J., Esmaiel H. (2022). Real signal DHT-OFDM with index modulation for underwater acoustic communication. IEEE J. Ocean. Eng..

[6] Guo J., Song S., Liu J., Chen H., Xu Y., Cui J.-H. (2024). Exploring applicable scenarios and boundary of MAC protocols: A MAC performance analysis framework for underwater acoustic networks. IEEE Trans. Mob. Comput..

[7] Nkenyereye L., Nkenyereye L., Ndibanje B. (2024). Internet of Underwater Things: A Survey on Simulation Tools and 5G-Based Underwater Networks. Electronics.

[8] Jehangir A., Ashraf S.M., Khalil R.A., Saeed N. (2024). ISAC-Enabled Underwater IoT Network Localization: Overcoming Asynchrony, Mobility, and Stratification Issues. IEEE Open J. Commun. Soc..

[9] Ma X., Wang B., Tian W., Ding X., Han Z. (2024). Strategic Game Model for AUV-Assisted Underwater Acoustic Covert Communication in Ocean Internet of Things. IEEE Internet Things J..

